# Prediabetes and insulin resistance in a population of patients with heart failure and reduced or preserved ejection fraction but without diabetes, overweight or hypertension

**DOI:** 10.1186/s12933-022-01509-5

**Published:** 2022-05-14

**Authors:** Tran Kim Son, Ngo Hoang Toan, Nguyen Thang, Huynh Le Trong Tuong, Hoang Anh Tien, Nguyen Hai Thuy, Huynh Van Minh, Paul Valensi

**Affiliations:** 1 Department of Internal Medicine, Can Tho University of Medicine and Pharmacy, Can Tho, Vietnam; 2 Science - Technology & External Relations Office, Can Tho University of Medicine and Pharmacy, Can Tho, Vietnam; 3 Cardiology Department, Can Tho Central General Hospital, Can Tho, Vietnam; 4 Department of Internal Medicine, Hue University of Medicine and Pharmacy, Hue University, Hue, Vietnam; 5 Unit of Endocrinology-Diabetology-Nutrition. Jean Verdier hospital, APHP, Sorbonne Paris Nord University, CINFO, CRNH-IdF, Bondy, France

**Keywords:** Heart failure, Heart failure with reduced ejection fraction, Heart failure with preserved ejection fraction, Oral glucose tolerance test, Prediabetes, Insulin resistance

## Abstract

**Background:**

The relationships between glucose abnormalities, insulin resistance (IR) and heart failure (HF) are unclear, especially regarding to the HF type, i.e., HF with reduced (HFrEF) or preserved (HFpEF) ejection fraction. Overweight, diabetes and hypertension are potential contributors to IR in persons with HF. This study aimed to evaluate the prevalence of prediabetes and IR in a population of Vietnamese patients with HFrEF or HFpEF but no overweight, diabetes or hypertension, in comparison with healthy controls, and the relation between prediabetes or IR and HF severity.

**Methods:**

We conducted a prospective cross-sectional observational study in 190 non-overweight normotensive HF patients (114 with HFrEF and 76 with HFpEF, 92.6% were ischemic HF, mean age was 70.1 years, mean BMI 19.7 kg/m^2^) without diabetes (neither known diabetes nor newly diagnosed by OGTT) and 95 healthy individuals (controls). Prediabetes was defined using 2006 WHO criteria. Glucose and insulin levels were measured fasting and 2 h after glucose challenge. IR was assessed using HOMA-IR and several other indexes.

**Results:**

Compared to controls, HF patients had a higher prevalence of prediabetes (63.2% vs 22.1%) and IR (according to HOMA-IR, 55.3% vs 26.3%), higher HOMA-IR, insulin/glucose ratio after glucose and FIRI, and lower ISIT0 and ISIT120 (< 0.0001 for all comparisons), with no difference for body weight, waist circumference, blood pressure and lipid parameters. Prediabetes was more prevalent (69.3% vs 53.9%, p = 0.03) and HOMA-IR was higher (p < 0.0001) in patients with HFrEF than with HFpEF. Among both HFrEF and HFpEF patients, those with prediabetes or IR had a more severe HF (higher NYHA functional class and NT-proBNP levels, lower ejection fraction; p = 0.04–< 0.0001) than their normoglycemic or non-insulinresistant counterparts, with no difference for blood pressure and lipid parameters.

**Conclusion:**

In non-diabetic non-overweight normotensive patients with HF, the prevalence of prediabetes is higher with some trend to more severe IR in those with HFrEF than in those with HFpEF. Both prediabetes and IR are associated with a more severe HF. The present data support HF as a culprit for IR. Intervention strategies should be proposed to HF patients with prediabetes aiming to reduce the risk of incident diabetes. Studies should be designed to test whether such strategies may translate into an improvement of further HF-related outcomes.

## Background

Heart failure (HF) is a leading cause of morbidity and mortality in Western countries. Its prevalence has been increasing, as well as its financial burden [1–3]. In recent cardiovascular outcome trials including patients with type 2 diabetes and a history of cardiovascular disease or several associated risk factors, the incidence of hospitalization for HF was comparable to the incidence of acute myocardial infarction [4]. The prevalence of patients with both HF and diabetes keeps increasing as the population is ageing [5] and diabetes is among the most common co-morbidities in HF patients, potentially increasing the risk of hospitalization and death [5]. Interestingly, HF with preserved ejection fraction (HFpEF) is currently the most frequent form of HF [6].

Glucose abnormalities often remain undiagnosed in HF patients [7] although the incidence of diabetes is high [8]. An exploratory analysis from the Dapagliflozin and Prevention of Adverse Outcomes in Heart Failure (DAPA-HF) trial looked at the incidence of diabetes (HbA1c ≥ 6.5% or initiation of a glucose-lowering agent) in the subgroup of patients with HF and reduced left ventricle ejection fraction (HFrEF), no prior history of diabetes and an HbA_1c_ level < 6.5% at baseline. In this subgroup, the incidence of newly-diagnosed diabetes was 7.1% in the placebo group over a median follow-up of 18 months [9].

Insulin resistance (IR) is frequent in HF patients, independently of an ischemic etiology [10, 11]. In a community cohort, IR was associated with incident HF and this relationship was not modified by BMI [12]. IR was also shown to be a predictor of death among HF patients without known diabetes [13]. IR has been frequently described among patients with HFrEF [13, 14]. Whether or not the prevalence of IR is different in patients with HFpEF has not been clearly established. A recent study on a small series showed that older patients (70–90 years) with either systolic HF or diastolic HF were more insulin resistant than same age healthy volunteers [14]. Another study suggested more severe IR in HFrEF patients than in HFpEF patients [15]. However, most studies looking at the association between HF and IR included few patients, and glucose status was not reported or patients with diabetes were not clearly excluded. While IR and HF may participate into a vicious circle, the precise relationship between IR and HF is an interesting but unsolved issue. In addition, obesity and hypertension are well-known risk factors for IR, type 2 diabetes but also HF [16, 17].

Thus, the relationships between glucose abnormalities, IR and HF need to be clarified. Known diabetes or newly diagnosed diabetes, obesity and hypertension should be considered as potential contributors to IR in the HF population. In addition, these relationships should be analyzed considering separately HFrEF and HFpEF.

The aim of this study was to evaluate the prevalence of prediabetes and IR in a population of non-diabetic non-overweight normotensive Vietnamese patients with HFrEF or HFpEF compared to healthy controls, and the relation of prediabetes and IR with HF severity.

## Methods

### Study design

We conducted a prospective cross-sectional observational study at Can Tho Central General Hospital, Vietnam, from April 2013 to May 2016, comparing consecutive patients with HF to healthy subjects.

This study was approved by the Institutional Review Board of this hospital. Information and data were secured. Patients and controls gave their consent for participation to the study. All the costs for lab tests were supported by the hospital research funding.

HF patients had been hospitalized for congestive episodes at least 6 months ago. Participants were in stable condition at the investigation time, with no change in treatments within this 6-months interval. The investigations were performed as outpatients or during hospitalization for clinical assessment and drug delivery. IR assessment and other biochemical measurements were performed a few days before echocardiography.

Healthy subjects were volunteers whose tests were performed in the hospital during an annual health check-up.

### Study population

#### Inclusion and exclusion criteria

Patients were included when they had a diagnosis of HF according to the 2016 European Society of Cardiology (ESC) guidelines [18]. The diagnosis of chronic HF was based on clinical symptoms and signs and confirmed with echocardiography showing evidence for an impaired myocardial function. According to guidelines [18], HFrEF was defined as LVEF ≤ 40% and HFpEF as LVEF ≥ 50%.

Patients with LVEF between 40 and 50% were excluded. We also excluded all patients with disorders or treatments which might potentially alter insulin sensitivity: (i) known diabetes, hypertension, chronic kidney disease defined as eGFR < 90 ml/min/1.73 m^2^, newly-diagnosed diabetes during Oral Glucose Tolerance Test (OGTT); (ii) BMI > 23 kg/m^2^ (the threshold for overweight diagnosis in Asian populations) or waist circumference > 90 cm for men,  > 80 cm for women [19]; (iii) current hormonal therapies (substitutive treatment for menopause, corticosteroids and beta-adrenoreceptor agonists).

Healthy controls were randomly selected by screening among healthy volunteers. They had similar age, gender, BMI, and other anthropometric characteristics and no history, symptoms or signs of cardiovascular disease including HF, no acute or chronic disease and no treatment susceptible to alter IR.

### Clinical assessment

All HF participants underwent a clinical comprehensive evaluation including an interview, complete physical examination, and echocardiography. HF was considered of ischemic cause when the patient had been previously diagnosed with coronary artery disease with no other explanation for HF.

In HF and control subjects, waist circumference was measured at the end of mild expiration, parallel to the floor using a supported tape. Measurement was made naked trunk, at the narrowest section, half-distance between the lower border of the costal margin and the iliac crest. Body weight and height were obtained with light clothing on. Body mass index was calculated using the weight (kg)/height (m)^2^ formula. Blood pressure was measured on both arms in the sitting position, and the average value was calculated.

### Biochemical measurements

Blood samples were collected after an overnight fast. They were immediately sent to the central hospital laboratory and processed for biochemical measurements.

#### Assessment of glucose status and insulin resistance

An OGTT was performed and plasma glucose and insulin levels were measured at fasting (G_0_ and I_0_) and 2 h after glucose intake (G_2_ and I_2_).

Three prediabetic conditions were considered, as defined by WHO 2006 criteria [20]: impaired fasting glucose (IFG) by G_0_ = 6.1–6.9 mmol/l (110–125 mg/dl), impaired glucose tolerance (IGT) defined by G_2_ = 7.8–11.1 mmol/l (140–200 mg/dl), or combined IFG and IGT. Normal glucose tolerance (NGT) was defined by G_0_ < 6.1 mmol/l (110 mg/dl) and G_2_ < 7.8 mmol/l (140 mg/dl).

Insulin resistance was assessed using several indexes: HOMA-IR index (HOmeostatic Model Assessment of Insulin Resistance: I_0_ × G_0_/22.55) [21] with a cut-off value defined by the highest quartile of the control group; QUICKI [QUantitative Insulin-sensitivity ChecK Index: 1/log(I_0_ + G_0_)] [22] with a cut-off value defined as the lowest quartile of the control group; and by I_0_, I_0_/G_0_, I_2_ and I_2_/G_2_ with cut-off values defined the respective mean + 1SD values of the control group. We also calculated additional insulin resistance indexes: fasting Insulin Sensitivity Index [ISIT0 = 10,000/(I_0_ × G_0_)], 2 h-Insulin Sensitivity Index [ISIT120 = 10,000/(I_2_ × G_2_)] [23] and Fasting Insulin Resistance Index [FIRI = (I_0_ × G_0_)/25)] [24].

#### Analytical methods

Plasma glucose, HbA1c, total cholesterol, triglycerides, HDL-cholesterol, and insulin were measured using HumaStar 600 (Wiesbaden, Germany). LDL-cholesterol was calculated using Friedwald formula. NT-proBNP was measured by electrochemiluminescence immuno-assay using Cobas E (Roche Diagnostics, USA).

### Statistical analyses

The normality of the data was assessed using the Kolmogorov–Smirnov test. Data are presented as mean ± standard deviation (SD) or as median [interquartile] if the distribution was not normal. Between-groups comparisons for continuous variables were performed by analyses of variance if normally distributed or by nonparametric tests if not. Between-groups comparisons for categorical parameters were performed by chi square tests. Odds ratios (OR) with 95% confidence intervals (CI) for the risk of IR in HF patients are reported. A p value < 0.05 was considered as statistically significant. Statistical analyses were carried out using SPSS statistical software version 22.0.

## Results

### Clinical characteristics of the study population

**Fig. 1:**
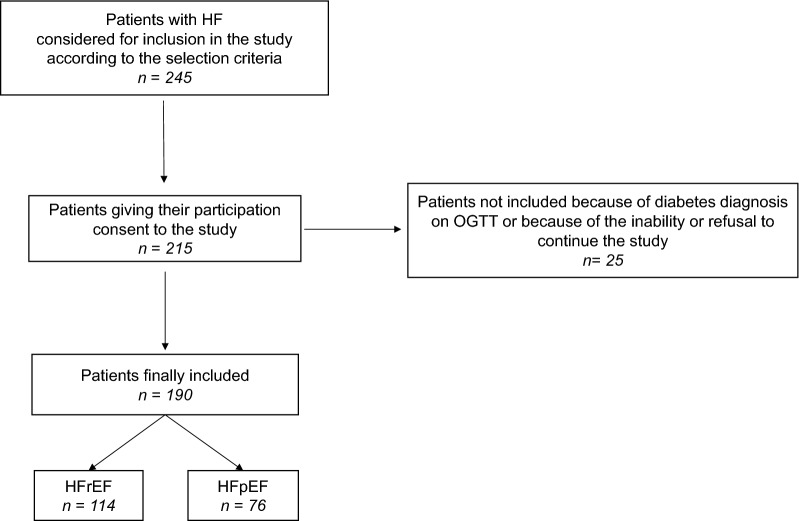
Flow chart of the study

Among 245 HF patients fulfilling the selection criteria, 215 gave their consent for participation to the study. Among them, 25 were either diagnosed with diabetes during OGTT or withdrew consent. Thus, the study population included 190 HF patients. Age range was 50 to 85 years, 114 patients had HFrEF and 76 HFpEF (Fig. 1). Baseline characteristics of these patients and of the 95 control subjects are shown in Table 1. Sex-ratio, age, BMI and waist circumference did not differ significantly between HF patients and controls. Among HF patients, these parameters did not differ significantly between those with HFrEF and those with HFpEF. Total and LDL cholesterol were higher in HFrEF patients. Cardiac ischemic disease was the main cause of HF (92.6%).

**Table 1:** Characteristics of the study population

	HF patients					
	Total HF	HFrEF	HFpEF	p*	Controls	p**
	(n = 190)	(n = 114)	(n = 76)		(n = 95)	
Gender (%)						
Male	95 (50)	55 (48.2)	40 (52.6)	0.813	46 (48.4)	0.802
Female	95 (50)	59 (51.8)	36 (47.4)		49 (51.6)	
Age (years)	70.1 ± 14.5	70.5 ± 14.0	69.5 ± 15.4	0.162	66.7 ± 14.7	0.064
BMI (kg/m^2^)	19.7 ± 1.5	19.8 ± 1.4	19.5 ± 1.6	0.202	19.9 ± 2.0	0.091
Waist circumference (cm)	74.4 ± 4.3	74.2 ± 4.2	74.6 ± 4.6	0.411	74.6 ± 4.3	0.743
Systolic BP (mmHg)	116.5 ± 15.4	117.5 ± 15.8	115.0 ± 14.7	0.233	114.3 ± 12.0	0.223
Diastolic BP (mmHg)	71.1 ± 10.4	71.3 ± 10.3	70.9 ± 10.7	0.646	71.7 ± 9.4	0.646
LVEF (%)	42.9 ± 14.4	32.2 ± 5.8	58.9 ± 6.3	< 0.001		
HF ischemic etiology (%)	176 (92.6)	110 (96.5%)	66 (86.8%)	0.013		
NYHA class						
II	32 (16.8)	9 (7.9)	23 (30.3)	< 0.0001		
III	150 (78.9)	97 (85.1)	53 (69.7)			
IV	8 (4.2)	8 (7.0)	0 (0)			
NT-pro-BNP (pg/ml)	9931 ± 7747	11,550 ± 8702	7504 ± 5215	< 0.0001		
HbA1c (%)	5.70 ± 0.50	5.71 ± 0.50	5.70 ± 0.51	0.81		
HbA1c ≥ 6.5% (%)	0	0	0			
G_0_ (mmol/l)	5.28 ± 0.79	5.37 ± 0.76	5.13 ± 0.82	< 0.0001	4.81 ± 0.81	< 0.0001
G_2_ (mmol/l)	8.07 ± 1.18	7.83 ± 1.14	6.93 ± 0.93	< 0.0001	7.13 ± 1.73	< 0.0001
I_0_ (µU/ml)	12.1 [5.3–23.7]	12.6 [5.5–26.4]	11.1 [4.5–21.7]	0.675	8.2 [5.1–13.0]	0.003
I_2_ (µU/ml)	90.3 [47.6–136.6]	95.0 [45.3–141.2]	73.6 [48.0–127.0]	0.054	50.0 [24.0–96.0]	< 0.0001
Total cholesterol (mmol/l)	4.17 ± 1.50	4.44 ± 1.66	3.77 ± 1.13	0.03		
Triglycerides (mmol/l)	1.57 ± 1.13	1.74 ± 1.33	1.32 ± 0.64	0.13		
LDL-cholesterol (mmol/l)	2.62 ± 0.98	2.78 ± 1.04	2.38 ± 0.82	0.05		
HDL-cholesterol (mmol/l)	1.01 ± 0.30	1.04 ± 0.31	0.97 ± 0.28	0.113		
Glycemic status (%)						
IFG alone	4 (2.1)	4 (2.1)	0	0.032	6 (6.3)	< 0.0001
IGT alone	94 (49.5)	64 (56.1)	30 (39.5)		15 (15.8)	
IGT + IFG	22 (11.6)	11 (9.6)	11 (14.5)		2 (2.1)	
NGT	70 (36.8)	35 (30.7)	35 (46.1)		72 (75.8)	
Medications (%)						
ACEIs/ARBs	88 (46.3)	70 (61.4)	18 (23.7)	< 0.0001		
Aldosterone antagonists	61 (32.1)	61 (53.5)	0 (0)	< 0.0001		
Beta-blockers	120 (63.2)	91 (79.8)	29 (38.2)	< 0.0001		
Diuretics	52 (27.4)	46 (40.4)	6 (7.9)	< 0.0001		
Ivabradine	40 (21.1)	36 (31.6)	4 (5.3)	< 0.0001		
Digoxin	13 (6.8)	13 (11.4)	0 (0)	0.002		
Antiplatelets	176 (92.6)	110 (96.5)	66 (86.8)	0.014		
Statin	159 (83.7)	98 (86.0)	61 (80.3)	0.310		
Nitrate	31 (16.3)	19 (16.7)	12 (15.8)	0.950		

*ACEIs/ARBs* angiotensin-converting-enzyme inhibitors/angiotensin II receptor blockers; *BP* blood pressure; *HF* heart failure; *HFrEF* and *HFpEF* HF with reduced and preserved ejection fraction; *BMI* body mass index; *G*_*0*_ and *I*_*0*_ plasma glucose and insulin levels measured fasting; *G*_*2*_ and *I*_*2*_ plasma glucose and insulin levels measured 2 h after glucose intake; *IFG* impaired fasting glucose; *IGT* impaired glucose tolerance; *NGT* normal glucose tolerance; *NT-pro-BNP* N-terminal pro Brain Natriuretic Peptide; *NYHA* New-York Heart Association

*p values for comparisons between HFrEF and HFpEF patients

**p for comparisons between the total HF group and the control group

Compared to HFpEF patients, HFrEF patients had higher NT-pro-BNP levels, were more often in NYHA functional class III or IV, and had more often HF from ischemic cause (Table 1).

### Glycemic status

Based on OGTT results, the prevalence of prediabetes was significantly higher in HF patients compared to control subjects (63.2% vs 24.2%, respectively; p < 0.0001). Among HF patients, the prevalence of IGT was significantly higher and the prevalence of NGT significantly lower (p < 0.0001) in patients with HFrEF compared to those with HFpEF (Table 1).

Compared to control subjects, G_0_ and G_2_, and I_0_ and I_2_ were higher in HF patients. HbA1c levels did not differ significantly, and none of the HF patients had an HbA1c level ≥ 6.5%. G_0_ and G_2_ were higher in HFrEF compared to HFpEF patients, without significant difference for HbA1c levels (Table 1).

### Prevalence of insulin resistance in patients with HFrEF and HFpEF

Most of the IR indexes differed significantly between HF patients and controls. HOMA-IR, I_2_/G_2_ and FIRI were higher, and ISIT0 and ISIT120 lower in HF patients compared to controls. Compared to HFpEF patients, HFrEF patients had higher HOMA-IR with a trend for higher FIRI and lower ISIT0 (Table 2).

**Table 2:** Comparisons of the indexes of insulin resistance in HF groups and in the control group

	HF patients			p	Controls	p*
	Total HF	HFrEF	HFpEF			
	(n = 190)	(n = 114)	(n = 76)		(n = 95)	
HOMA-IR	4.10 ± 3.70	4.51 ± 3.89	3.49 ± 3.31	< 0.0001	1.86 ± 1.01	< 0.0001
I_0_/G_0_	2.34 [6.40–17.76]	2.37 [1.10–4.75]	2.12 [0.89–4.05]	0.882	1.80 [1.10–2.60]	0.146
I_2_/G_2_	11.18 [6.40–17.76]	11.60 [6.46–19.56]	9.76 [6.29–16.08]	0.183	7.10 [3.60–13.00]	< 0.0001
QUICKI	0.63 ± 0.25	0.61 ± 0.27	0.65 ± 0.23	0.416	0.65 ± 0.11	0.452
ISIT0	8.53 [4.02–21.45]	7.89 [3.50–20.17]	9.74 [5.03–27.03]	0.079	14.19 [9.03–22.05]	< 0.0001
ISIT120	0.76 [0.47–1.63]	0.70 [0.44–1.68]	0.96 [0.53–1.63]	0.213	1.72 [0.83–3.73]	< 0.0001
FIRI	2.60 [1.04–5.53]	2.81 [1.10–6.36]	2.28 [0.82–4.41]	0.079	1.57 [1.01–2.46]	< 0.0001

*FIRI* fasting insulin resistance index; *I*_*0*_*/G*_*0*_ plasma insulin/glucose ratio measured at fasting; *I*_*2*_*/G*_*2*_ plasma insulin/glucose ratio measured 2 h after glucose intake; *HOMA-IR* homeostatic model assessment of insulin resistance; *ISIT0* fasting insulin sensitivity index; *ISIT120* 2 h-insulin sensitivity index; *QUICKI* quantitative insulin-sensitivity check index

p: comparisons between HFrEF and HFpEF

p*: comparisons between the total HF group and control group

We then considered the cut-off values of IR indexes on the basis of values in the control group. The cut-off values were 2.53 (highest quartile) for HOMA-IR index; 0.33 (lowest quartile) for QUICKI and the mean + 1SD for I_0_/G_0_ and I_2_/G_2_ indexes in the control group. The prevalence of IR as defined by values over the cut-off values according to HOMA-IR was significantly higher in patients with HFrEF and HFpEF (58.8% and 50.0%, respectively) than in the control group (p < 0.001). Based on the other IR indexes including I_0_/G_0_, I_2_/G_2_ and QUICKI, the prevalence of IR in HFrEF and HFpEF groups was 30.7% to 60.5% and 26.3% to 50%, respectively, without significant difference between the two groups (Table 3).

**Table 3:** Percentages of patients with insulin resistance indexes above (for HOMA-IR, I_0_/G_0_, I_2_/G_2_) or below (for QUICKI) the respective cut-off values

Index	Cut-off Value	HF groups			p	Control group (%)	p*
		Total HF (%)	HFrEF (%)	HFpEF (%)			
HOMA-IR	2.53	55.3	58.8	50.0	0.236	26.3	< 0.0001
I_0_/G_0_	3.81	30.0	31.6	27.6	0.563	13.7	0.008
I_2_/G_2_	14.54	28.9	30.7	26.3	0.516	17.9	0.103
QUICKI	0.33	56.3	60.5	50.0	0.153	30.5	< 0.0001

*HOMA-IR* homeostatic model assessment of insulin resistance; *I*_*0*_*/G*_*0*_ plasma insulin/glucose ratio measured at fasting; *I*_*2*_*/G*_*2*_ plasma insulin/glucose ratio measured 2 h after glucose intake; *QUICKI* quantitative insulin-sensitivity check index

p: comparisons between HFrEF and HFpEF groups

p*: comparisons between the total HF group and the control group

### Likelihood of IR in HF patients

Compared to controls, HFrEF patients had a highly significant likelihood of IR according to these cut-off values for all the indexes with very high OR, especially for HOMA index and QUICKI. The same significant trend (except for I_2_/G_2_) was observed in HFpEF patients**.** Meanwhile, there was no significant difference between HFrEF and HFpEF populations for the risk of IR based on any of these indexes (Table 4).

**Table 4:** Odds ratios for the risk of IR in HFrEF and HFpEF groups compared to controls and between HF groups

Index	OR (CI 95%)		
	HFrEF vs controls	HFpEF vs controls	HFrEF vs HFpEF
HOMA-IR	**4.00 (2.21–7.76) *p* < *0.001***	**2.81 (1.48–5.32) *p* < *0.001***	1.43 (0.71–2.56) *p* > *0.05*
I_0_/G_0_	**2.91 (1.44–5.90) *p* < *0.05***	**2.41 (1.11–5.21) *p* < *0.05***	1.21 (0.64–2.29) *p* > *0.05*
I_2_/G_2_	**2.03 (1.05–3.93) *p* < *0.05***	1.64 (0.79–3.41) *p* > *0.05*	1.24 (0.65–2.37) *p* > *0.05*
QUICKI	**3.49 (1.96–6.21) *p* < *0.001***	**2.76 (1.22–4.26) *p* < *0.01***	1.53 (0.57–1.09) *p* > *0.05*

Numbers marked in bold indicate a significant OR (p < 0.05)

*HOMA-IR* homeostatic model assessment of insulin resistance; *I*_*0*_*/G*_*0*_ plasma insulin/glucose ratio measured at fasting; *I*_*2*_*/G*_*2*_ plasma insulin/glucose ratio measured 2 h after glucose intake; *QUICKI* quantitative insulin-sensitivity check index; 95% *CI* confidence interval; *OR* odd ratio

### Comparison between HF patients with or without prediabetes and with or without IR

Among HFrEF and HFpEF patients considered separately, those with prediabetes were more often in high NYHA class and had higher NT-proBNP level and lower LVEF than those with NGT. IR was also more pronounced in those patients as shown by significantly different values for all the IR indexes and higher glycemic levels, with no other difference for clinical or biological parameters nor for the etiology of HF (Table 5). Similar trends were found in patients with IR (as defined by HOMA-IR ≥ 2.53) compared to those without, with no significant difference for BMI, waist circumference and lipid parameters. Nevertheless, more patients with dysglycemia were found among patients with IR (Table 6).

**Table 5:** Comparisons of HFrEF and HFpEF patients by glycemic status, i.e., with either prediabetes or normoglycemia

	HFrEF			HFpEF		
	Prediabetes	NGT	p values	Prediabetes	NGT	p values
	(n = 79)	(n = 35)		(n = 41)	(n = 35)	
Gender (%)						
Male	37 (46.8)	18 (51.4)	0.651	24 (58.5)	16 (45.7)	0.264
Female	42 (53.2)	17 (48.6)		17 (41.5)	19 (54.3)	
Age (years)	70.8 ± 13.7	69.9 ± 14.8	0.775	69.8 ± 15.7	69.2 ± 15.3	0.865
Waist circumference (cm)	74.5 ± 3.9	73.7 ± 4.9	0.391	75.2 ± 4.5	73.8 ± 4.6	0.197
BMI (kg/m^2^)	19.7 ± 1.4	19.9 ± 1.3	0.438	19.4 ± 1.6	19.5 ± 1.6	0.852
Systolic BP (mmHg)	117.7 ± 16.1	117.0 ± 15.4	0.833	114.0 ± 13.0	116.3 ± 16.5	0.503
Diastolic BP (mmHg)	71.2 ± 10.3	71.3 ± 10.4	0.972	70.6 ± 9.1	71.3 ± 12.4	0.762
LVEF (%)	30.9 ± 6.2	35.2 ± 5.6	< 0.0001	57.6 ± 6.1	60.5 ± 6.2	0.044
HF ischemic etiology (%)	76 (96.2)	34 (97.1)	0.801	34 (82.9)	33 (91.4)	0.274
NYHA class						
II	2 (2.5)	7 (20.0)	< 0.001	7 (17.1)	16 (45.7)	0.007
III	69 (87.3)	28 (80)		34 (82.9)	19 (54.3)	
IV	8 (10.1)	0				
NT-pro-BNP (pg/ml)	13,138 ± 9088	7964 ± 6566	0.003	9032 ± 5296	5715 ± 4569	0.005
HbA1c (%)	5.71 ± 0.50	5.72 ± 0.50	0.885	5.64 ± 0.56	5.75 ± 0.46	0.356
Total cholesterol (mmol/l)	4.60 ± 1.87	4.08 ± 1.02	0.127	3.55 ± 0.95	4.05 ± 1.27	0.056
Triglycerides (mmol/l)	1.82 ± 1.47	1.57 ± 0.98	0.358	1.28 ± 0.61	1.39 ± 0.69	0.461
LDL-cholesterol (mmol/l)	2.86 ± 1.14	2.62 ± 0.80	0.271	2.24 ± 0.73	2.54 ± 0.91	0.120
HDL- cholesterol (mmol/l)	1.05 ± 0.32	1.04 ± 0.33	0.942	0.95 ± 0.30	0.99 ± 0.28	0.518
Glycemic status						
NGT, n (%)	0	35 (100%)	< 0.0001	0	35 (100)	< 0.0001
IGT alone, n (%)	64 (81)	0		30 (73.2)	0	
IFG alone, n (%)	4 (5.1)	0		0	0	
IGT + IFG, n (%)	11 (13.9)	0		11 (26.8)	0	
G_0_ (mmol/l)	5.57 ± 0.74	4.95 ± 0.64	< 0.0001	5.43 ± 0.83	4.80 ± 0.68	< 0.001
G_2_ (mmol/l)	8.84 ± 0.81	6.88 ± 0.62	< 0.0001	8.70 ± 0.72	6.81 ± 0.54	< 0.0001
I_0_/G_0_	3.15 [1.92–6.16]	1.06 [0.53–2.00]	< 0.0001	2.82 [1.48–4.85]	1.32 [0.57–3.32]	0.066
I_2_/G_2_	13.56 [10.10–22.06]	7.80 [2.85–12.05]	0.007	11.28 [8.11–26.29]	7.70 [4.10–12.46]	0.021
QUICKI	0.53 ± 0.16	0.79 ± 0.36	< 0.0001	0.56 ± 0.14	0.74 ± 0.27	< 0.0001
ISIT0	4.89 [2.94–10.13]	21.38 [10.65–40.21]	< 0.0001	7.42 [3.65–14.89]	19.25 [6.05–43.27]	0.001
ISIT120	0.56 [0.33–0.76]	2.23 [0.81–4.28]	< 0.0001	0.66 [0.30–1.05]	1.55 [0.90–3.39]	< 0.0001
FIRI	4.54 [2.19–7.56]	1.04 [0.55–2.09]	< 0.0001	2.99 [1.51–6.09]	1.15 [0.51–3.68]	0.001

*BMI* body mass index; *BP* blood pressure; *FIRI* fasting insulin resistance index; *G*_*0*_ and *I*_*0*_ plasma glucose and insulin levels measured fasting; *G*_*2*_ and *I*_*2*_ plasma glucose and insulin levels measured 2 h after glucose intake; *HF* heart failure; *HFrEF* and *HFpEF* HF with reduced and preserved ejection fraction; *HOMA-IR* homeostatic model assessment of insulin resistance; *IFG* impaired fasting glucose; *IGT* impaired glucose tolerance; *ISIT0* fasting insulin sensitivity index; *ISIT120* 2 h-insulin sensitivity index; *NGT* normal glucose tolerance; *NT-pro-BNP* N-terminal pro Brain Natriuretic Peptide; *NYHA* New-York Heart Association; *QUICKI* quantitative insulin-sensitivity check index

**Table 6:** Comparisons of HFrEF and HFpEF patients by insulin resistance status, i.e., with HOMA ≥ 2.53 or < 2.53

	HFrEF			HFpEF		
	HOMA ≥ 2.53	HOMA < 2.53	p values	HOMA ≥ 2.53	HOMA < 2.53	p values
	(n = 67)	(n = 47)		(n = 38)	(n = 38)	
Gender (%)						
Male	31 (46.3)	24 (51.1)	0.614	17 (44.7)	23 (60.5)	0.168
Female	36 (53.7)	23 (48.9)		21 (55.3)	15 (39.5)	
Age (years)	70.3 ± 13.2	70.8 ± 15.1	0.849	71.1 ± 13.7	67.9 ± 17.0	0.367
BMI (kg/m^2^)	19.8 ± 1.5	19.8 ± 1.1	0.915	19.7 ± 1.6	19.3 ± 1.7	0.294
Waist circumference (cm)	74.7 ± 3.9	73.5 ± 4.5	0.138	75.0 ± 4.5	74.2 ± 4.7	0.441
Systolic BP (mmHg)	116.9 ± 14.4	118.3 ± 17.7	0.661	118.3 ± 13.1	111.8 ± 15.6	0.052
Diastolic BP (mmHg)	71.1 ± 9.2	71.5 ± 11.8	0.817	73.5 ± 9.59	68.3 ± 11.3	0.034
LVEF (%)	29.6 ± 5.9	36.0 ± 3.1	< 0.0001	54.5 ± 3.2	63.3 ± 5.5	< 0.0001
HF ischemic etiology (%)	65 (97.0)	45 (95.7)	0.717	32 (84.2)	34 (89.5)	0.497
NYHA class						
II	1 (1.5)	8 (17.0)	< 0.001	3 (7.9)	20 (52.6)	< 0.0001
III	58 (86.6)	39 (83.0)		35 (92.1)	18 (47.4)	
IV	8 (11.9)	0 (0)		0 (0)	0	
NT-pro-BNP (pg/ml)	15,046 ± 9104	6565 ± 4891	< 0.0001	10,857 ± 4823	4151 ± 2962	< 0.0001
HbA1c (%)	5.68 ± 0.51	5.75 ± 0.49	0.487	5.66 ± 0.56	5.72 ± 0.48	0.588
Total cholesterol (mmol/l)	4.44 ± 1.25	4.44 ± 2.14	0.995	3.80 ± 1.07	3.76 ± 1.20	0.88
Triglycerides (mmol/l)	1.89 ± 1.33	1.53 ± 1.34	0.163	1.46 ± 0.71	1.19 ± 0.55	0.075
LDL-cholesterol (mmol/l)	2.78 ± 0.89	2.80 ± 1.25	0.937	2.44 ± 0.77	2.31 ± 0.89	0.518
HDL- cholesterol (mmol/l)	1.01 ± 0.30	1.10 ± 0.32	0.214	0.97 ± 0.29	0.98 ± 0.29	0.937
Glycemic status						
NGT, n (%)	7 (10.4)	28 (59.6)	< 0.0001	12 (31.6)	23 (60.5)	0.011
IGT alone, n (%)	49 (73.1)	15 (31.9)		19 (50)	11 (28.9)	
IFG alone, n (%)	2 (3.0)	2 (4.3)		0	0	
IGT + IFG, n (%)	9 (13.4)	2 (4.3)		7 (18.4)	4 (10.5)	
G_0_ (mmol/l)	5.56 ± 0.68	5.12 ± 0.81	0.002	5.33 ± 0.78	4.95 ± 0.83	0.039
G_2_ (mmol/l)	8.78 ± 0.90	7.47 ± 1.11	< 0.0001	8.30 ± 1.12	7.36 ± 0.97	< 0.0001
I_0_/G_0_	3.95 [2.80–7.30]	0.92 [0.55–1.25]	< 0.0001	4.03 [3.20–5.05]	0.93 [0.50–1.41]	< 0.0001
I_2_/G_2_	15.17 [11.60–24.36]	6.30 [2.85–9.80]	< 0.0001	15.95 [10.52–27.41]	6.33 [3.24–8.52]	< 0.0001
QUICKI	0.48 ± 0.05	0.80 ± 0.33	< 0.0001	0.49 ± 0.05	0.80 ± 0.22	< 0.0001
ISIT0	4.09 [2.73–6.80]	21.89 [13.02–38.94]	< 0.0001	5.06 [3.43–7.26]	26.79 [13.63–43.27]	< 0.0001
ISIT120	0.50 [0.30–0.67]	2.06 [0.92–3.53]	< 0.0001	0.57 [2.90–0.81]	1.63 [1.04–3.32]	< 0.0001
FIRI	5.43 [3.26–8.14]	1.02 [0.57–1.71]	< 0.0001	4.39 [3.06–6.47]	0.83 [0.51–1.63]	< 0.0001

*BMI* body mass index; *BP* blood pressure; *FIRI* fasting insulin resistance index; *G*_*0*_ and *I*_*0*_ plasma glucose and insulin levels measured fasting; *G*_*2*_ and *I*_*2*_ plasma glucose and insulin levels measured 2 h after glucose intake; *HF* heart failure; *HFrEF* and *HFpEF* HF with reduced and preserved ejection fraction; *HOMA-IR* homeostatic model assessment of insulin resistance; *IFG* impaired fasting glucose; *IGT* impaired glucose tolerance; *ISIT0* fasting insulin sensitivity index; *ISIT120* 2 h-insulin sensitivity index; *NGT* normal glucose tolerance; *NT-pro-BNP* N-terminal pro Brain Natriuretic Peptide; *NYHA* New-York Heart Association; *QUICKI* quantitative insulin-sensitivity check index

## Discussion

The most important findings of this study are that in a Vietnamese population of patients with HF and no diabetes, overweight or hypertension, (i) the prevalence of prediabetes and IR is high, (ii) prediabetes and IR are associated with a higher degree of HF, (iii) both findings affect similarly HFrEF and HFpEF patients.

### Prediabetes, an underdiagnosed condition and a marker of worse cardiac function in patients with HFrEF or HFpEF

It is commonly agreed that 20–30% of patients with HFrEF have had a previous diagnosis of diabetes. European guidelines on diabetes, prediabetes and cardiovascular disease recommend performing an OGTT in all patients with CVD and no known diabetes [25]. This recommendation is supported by data in patients with coronary disease [26]. In addition, the presence of prediabetes is associated with an increased risk of cardiovascular events and incident type 2 diabetes. In a series of stable subjects with prediabetes who underwent elective coronary angiography, the presence of subclinical myocardial necrosis as detected by high-sensitivity cardiac troponin T was reported to be prevalent and portend heightened long-term adverse cardiovascular event risk [27]. Thus, this marker may help to stratify cardiovascular risk in prediabetic subjects. Regarding the risk of HF, in the Multi-Ethnic Study of Atherosclerosis (MESA) HbA_1c_ and fasting plasma glucose in the diabetes but not in the prediabetes range were each associated with higher risks of incident hospitalization for HF (HFpEF or HFrEF) [28]. Stratifying the risk of incident type 2 diabetes in prediabetic subjects using new markers [29] may also be helpful.

The reports on the frequency of glucose abnormalities in non-ischemic HF patients are scarce and included only a small number of participants [30, 31]. In a series of 227 HFrEF patients who had an OGTT, 23% were classified as having IGT and 18% as having newly diagnosed diabetes, and similar percentages were found in patients with either ischemic or non-ischemic HF [7]. In a subanalysis of the SUPPORT trial including 535 HF patients without known diabetes (18% with HFrEF and 62% with HFpEF), IGT was found in 23% of those participants [32]. However, there was no published study comparing adequately the prevalence of newly diagnosed glucose disorders in HFrEF and HFpEF patients. In our study, we were careful to exclude not only patients with previously diagnosed diabetes but also those diagnosed with diabetes according to OGTT and HbA1c was < 6.5% in all the participants. Furthermore, we excluded potential confounders such as overweight, hypertension, impaired renal function and current hormonal therapies that could have changed glucose metabolism. Compared with controls who had the same sex-ratio and BMI but were slightly younger, the prevalence of prediabetes (mostly IGT) during OGTT was more than doubled, reaching 63.2%, in HF participants. Noteworthy, if only fasting plasma glucose levels had been measured, 94 participants with IGT alone out of the 120 patients with prediabetes (78%) would have been undiagnosed.

Prediabetes affected slightly more patients with HFrEF than those with HFpEF (66% vs 54% respectively). When HFrEF and HFpEF patients were taken separately, those with prediabetes had more severe HF with a higher NYHA functional class and higher plasma NT-proBNP levels. Ultrasound-measured LVEF was lower in participants with prediabetes compared to their normoglycemic counterparts while age, gender, BMI, waist circumference, blood pressure, lipid levels and the proportion of ischemic HF did not differ significantly.

In HF patients, a subgroup analysis of CHARM study reported a remarkably high prevalence of dysglycemia as detected by HbA1c ≥ 6.0% irrespective of ejection fraction phenotype and an association between dysglycemia and a higher risk of adverse clinical outcomes [33]. However, HbA1c ≥ 6.0% is not currently recognized as a cut-off point for dysglycemia and does not allow a clear diagnosis of glycemic status. The survival curve for HF patients with IGT or newly-diagnosed diabetes on OGTT was reported to be in intermediate positions between the curve for NGT and the one for previously diagnosed diabetes, with similar mortality rates for ischemic and non-ischemic HF [7]. In line with that study, our results showing that prediabetes is associated with worse cardiac function in HFrEF and HFpEF highlights the use of OGTT in risk stratification. Further studies should investigate whether or not interventions in patients with prediabetes and more specifically with IGT, may slow or reverse HF progression and related outcomes.

### Insulin resistance, a highly prevalent disorder associated with worse cardiac function in patients with HFrEF or HFpEF

In our study, various indexes were used to assess IR and IR was more pronounced in HF patients compared to healthy controls with a trend for more pronounced IR in HFrEF compared to HFpEF patients. When considering the cut-off values of IR indexes in the control group, the prevalence of IR was doubled in HF patients compared to controls. However, the prevalence of IR was close in both HF groups (58.8% and 50% according to HOMA-IR index, respectively) with no significant difference between HF groups for the risk of IR using several IR indexes. Our results are in line with previous studies reporting a high prevalence of IR among often overweight patients with HFrEF [13, 14, 34]. Noteworthy, cardiac ischemic disease was form far the most frequent cause of HF (92.6%) in our population, and that could explain, at least partly, the high prevalence of prediabetes and IR we report. Only a few publications compared IR in HFrEF and HFpEF patients and usually on a limited number of patients. Our results are consistent with two previous studies showing no statistically significant difference for HOMA-IR in HFrEF and HFpEF patients [14, 34]. In a recent study including only 60 HF patients, fasting and dynamic measures of IR were performed in non-diabetic individuals with stable ambulatory HFrEF or HFpEF and showed more severe IR in patients with HFrEF on the dynamic tests [15]. Importantly, in our study, the high prevalence of IR was shown in both HFrEF and HFpEF patients and for the first time after exclusion not only of the patients with previously diagnosed diabetes but also of those with newly diagnosed diabetes on OGTT and after exclusion of patients with overweight or hypertension and of other confounding factors. Furthermore, while most reports used only the HOMA-IR index to assess IR, our study demonstrates that combining this index with other indirect indexes contributes to identify IR risk in patients with HF. Specifically, QUICKI index and I_0_/G_0_ were relevant parameters to identify this risk and hence their use could be suggested in IR assessment. Further studies in larger populations will be useful to precise incremental insights of IR differences between HFrEF and HFpEF patients.

We did not observe any significant difference in metabolic syndrome components according to the presence or absence of IR, except for dysglycemia which was more prevalent in participants with IR. Whether IR is a consequence, or a cause of HF is unclear. As IR was highly prevalent despite normal body weight and no excess in abdominal adiposity and was not associated with pronounced metabolic disorders, IR was more likely the consequence rather than the cause of HF and this might secondarily lead to dysglycemia. The high incidence of new diabetes in HF patients is consistent with this hypothesis [9]. However, the glycemic alterations induced by IR, ranging from mild dysglycaemia to diabetes, may conversely increase the risk of HF and HF progression [5, 7]. How IR develops and worsens in HF patients is not well-understood, but IR may derive from many factors: enhanced sympathetic activation, loss of skeletal muscle mass, endothelial dysfunction, forced sedentary lifestyle due to reduced cardiac output and increased fatigability, a potential effect of increased circulating cytokines, and molecular mechanisms [6, 35–42]. The main mechanism involves probably ‘neurohormone hypothesis’ [43, 44]. Humoral neural stimuli are enhanced in both HFrEF and HFpEF, leading to an activation of the sympathetic nervous system and subsequently raised catecholamine levels. Catecholamine increase contributes to numerous adverse metabolic effects: negative impacts on insulin signaling and glucose utilization in skeletal muscle, reduction of insulin secretion, stimulation of hepatic gluconeogenesis and glycogenolysis, as well as elevated concentration of circulating free fatty acids as a result of increased adipocyte lipolysis [39]. All those mechanisms ultimately induce IR. However, the mechanism of catecholamine increase and the catecholamine levels are different in HFrEF and HFpEF. Catecholamine levels were reported to be higher in HFrEF compared to HFpEF. The increase in catecholamines may result from the activation of humoral neural stimuli secondary to the reduction in cardiac output in HFrEF and to alterations in left ventricular relaxation in HFpEF [11].

IR was reported to be an independent predictor of death among non-diabetic patients with HF [13], suggesting that impaired insulin sensitivity may play a role in the pathophysiology of HF progression. Some studies showed that the degree of IR correlated with the NYHA functional class of HF [34], the LVEF [13] and the peak oxygen consumption during a stress test [10, 13, 37]. In our study, when HFrEF and HFpEF patients were taken separately, participants with IR rather belonged to the highest NYHA functional classes and had higher NT-proBNP levels and lower LVEF compared to their counterparts without IR. This was observed after exclusion of the potential role of diabetes, overweight, hypertension or impairment of renal function, and whether or not patients had ischemic HF noting that ischemic HF was equally distributed in patients with or without IR. Furthermore, there was a trend for a more pronounced difference in HF severity according to the presence or absence of IR than according to the presence or absence of prediabetes (Tables 5 and 6). Thus, IR should also be considered for risk stratification in both HFrEF and HFpEF patients. Various mechanisms could explain how IR may alter cardiac function. In the presence of IR, the myocardium uses more free fatty acids and less glucose [45], and that increases heart vulnerability to ischemia and pressure load. Hyperinsulinemia increases sodium and fluid retention, enhances sympathetic nervous system activity [42] and may favor cardiac remodeling [46]. However, whether or not IR can be taken as a target for HF therapy remains uncertain. The Tayside observational study suggests that metformin may be beneficial in patients with HF and diabetes. This needs to be verified by a prospective clinical trial [47].

Finally, it is important to characterize glycemic and IR status in HF patients without previously known diabetes as it seems possible to reduce the incidence of diabetes in this population. A recent paper showed that compared with a strategy of general health education, a lifestyle intervention strategy can reverse glucose levels to normoglycemia in individuals with prediabetes [48]. In the HFrEF population included in the DAPA-HF trial, dapagliflozin treatment induced a 32% reduction in diabetes incidence and this effect was mainly driven by participants with prediabetes at baseline (HbA_1c_ 5.7–6.4%) [9]. The potential benefit of sodium glucose cotransporter 2 (SGLT2)-inhibitors in diabetes prevention needs confirmation in trials of longer duration. It looks crucial as DAPA-HF participants who developed diabetes had a higher subsequent mortality than those who did not [9]. This additional benefit of SGLT2 inhibitors on diabetes prevention in HF patients could be reinforced in the presence of prediabetes and/or IR.

### Strengths and limitations

Our study has major strengths. This was a prospective study including a reasonably large population of HF patients with either reduced or preserved left ventricle ejection fraction but a unique study design aiming to characterize glycemic and IR status in the total population. The population was relatively homogeneous due to the strict exclusion of patients with known or newly detected diabetes, hypertension or overweight and other factors that could have interfered with OGTT results or IR assessment. We were thus able to detect early stages of IR. The study has also some limitations. First, the study center was in Vietnam and the participants had a rather low BMI, around 20 kg/m^2^ in means: our findings may not be generalizable to other populations. In a previous study we showed that in non-overweight Vietnamese people, essential hypertension was associated with IR [49]. Overweight has been shown to be associated with a survival benefit in HF patients. Recent reports discussed a balanced reappraisal of this “obesity paradox” [50, 51]. Lean body weight in HF patients might result from weight loss associated with severe HF grade. However, our patients were in stable condition without evidence of recent weight loss. Future studies in non-overweight patients should take into account more accurately recent changes in body weight and carry out body composition evaluation. Second, the high prevalence of IR among our patients might be due to the high proportion of NYHA functional class III to IV (in 83% of the participants) and to the large predominance of ischemic HF. The association of IR with HF independently of the above-mentioned confounding factors needs to be confirmed in a population with other HF etiologies. Third, the cross-sectional design of the study prevents us from evaluating the impact of glycemic status and IR on HF-related outcomes. Fourth, HF patients were receiving different therapies, including renin angiotensin aldosterone system blockers, beta-blockers and diuretics, which may impact glucose metabolism. However, these treatments did not differ between patients with or without prediabetes or IR (data not shown).

## Conclusion

The present data show that in Vietnam, in a population of participants with HF but no diabetes, overweight or hypertension, the prevalence of prediabetes and of IR is high in those with HFpEF as in those with HFrEF. Both prediabetes and IR are associated with more severe HF. This study contributes to better define the early stages of IR in HF patients with no metabolic disorders except mild hyperglycaemia and underlines the need to better assess all HF patients for their metabolic profile, including those without overweight. The study also provides arguments for HF as the culprit for IR. These interactions between HF, IR and prediabetes might carry prognostic and therapeutic implications. Intervention strategies should be proposed to HF patients with prediabetes aiming to reduce the risk of incident diabetes. Studies should be designed to test whether such strategies may translate into an improvement of further HF-related outcomes.

## Data Availability

The datasets generated during and/or analyzed during the current study are available from the corresponding author on reasonable request.

## Abbreviations

BMI: Body mass index
EF: Ejection fraction
FIRI: Fasting insulin resistance index
HF: Heart failure
HFpEF: Heart failure with preserved left ventricle ejection fraction
HFrEF: Heart failure with reduced left ventricle ejection fraction
HOMA-IR: Homeostatic model assessment of insulin resistance
IFG: Impaired fasting glucose
IGT: Impaired glucose tolerance
IR: Insulin resistance
ISIT0: Fasting insulin sensitivity index
ISIT120: 2hH-insulin sensitivity index
NGT: Normal glucose tolerance
NT-proBNP: N-terminal pro Brain Natriuretic Peptide
NYHA: New-York Heart Association
OGTT: Oral glucose tolerance test
QUICKI: Quantitative insulin-sensitivity check index
